# A Case of Appendicitis with Unusual Sequelae1Read at the thirtieth annual meeting of the Medical Association of Central New York, held at Buffalo, October 19, 1897.

**Published:** 1898-05

**Authors:** Nathan Jacobson

**Affiliations:** Syracuse, N. Y., Professor of clinical surgery in Medical Department of Syracuse University; surgeon to St. Joseph’s Hospital, Syracuse, N. Y.


### A CASE OF APPENDICITIS WITH UNUSUAL SEQUELAE[1]

BY NATHAN JACOBSON, M. D., Syracuse, N. Y.,
Professor of clinical surgery in Medical Department of Syracuse University; surgeon to St. Joseph's Hospital, Syracuse, N. Y.

LET me present, as concisely as I can, the surgical history of a patient who has been under my observation since January 16, 1897:

His occupation had been that of a farmer. He is unmarried; 28 years of age. On September 12, 1896, at the request of his physician, Dr. Badgeley, he was operated upon at his home, at Fayetteville, N. Y., by the late Dr. Magee, of Syracuse, assisted by Dr. Elsner. I am indebted to the latter for the notes of that operation. At this time, the sixth day of his sickness, they found his features pinched, his skin bluish-red, his extremities cold, he hiccoughed constantly, vomited a greenish-yellow fluid. The abdomen was enormously distended and tympanitic. A large area of flatness occupied the right iliac and hypogastric regions. His temperature was 103°; pulse, 140; respirations, 19, reduced in frequency because of morphia narcosis. Dr. Magee made an incision along the right semilunar line. Upon reaching the peritoneal cavity the intestines were found glued together with a yellowish-gray exudate; the cecum and appendix seemed gangrenous. The appendix could be but partially seen. One quart of fetid pus escaped. The apparently gangrenous area in the neighborhood of the appendix was about three by four inches. Gauze was carried in all directions and several yards of it used. The operation lasted an hour and a half; the patient hiccoughed throughout the entire time.

---

1. Read at the thirtieth annual meeting of the Medical Association of Central New York, held at Buffalo, October 19, 1897.

We will all admit it to be surprising that so advanced a condition of septic invasion should have been relieved by a surgical measure, and that there was little at the time to warrant the prediction that the patient could recover. He remained under the care of Drs. Badgeley and Magee until the latter's death, October 29, 1896. From this date until he saw me, which, as stated, was January 16th, the local physician cared for him. When presented to me there was a sinus on the right side. A probe introduced into it recognised at once the presence of several stony hard masses. The sinus led into the right iliac fossa. Two weeks prior he had had some fever and a swelling was discovered in the left iliac fossa, just above Poupart's ligament. Here a tumor was present of perhaps the size of a hen's egg. An attempted examination per rectum caused so much pain that it was not completed. On January 18th a tupelo tent was placed in the sinus. After dilatation was effected a concretion was found lying about two inches from the surface and removed with the aid of a Volkman spoon. It had a circumference of 27 mm. and was 1½ cm. in length. This was done painlessly under cocaine.

After this, for a few days, the morning temperature remained normal, but there was a slight evening rise. On January 23, 1897, the temperature being 101½°, I made, under ether, an incision 2½ inches in length, parallel with Poupart's ligament, over the swelling on the left side. The hard mass was found to be made up of infiltrated tissue with its center broken down and containing a quantity of pus. It was freely curetted with a Volkman spoon. A probe could be carried into it downward and backward, apparently traveling close to the inner surface of the pelvis for a distance of about eight inches. Gauze was introduced into its depth and the wound partly closed. On the right side the sinus was further opened and an attempt made to reach its bottom, and although the probe entered very deeply there could be no certainty as to its depth. This operation disposed of the high temperature for a time. Only occasionally did the thermometer register 100°. Another fecal concretion was removed February 8, 1897. No improvement seemed to appear in the right-sided sinus, although the abscess cavity in the left side healed quite promptly.

During the early days of March, 1897, there was a further increase of temperature; a free discharge of pus persistently appeared on the right side, giving evidence of deeper disturbance. All of this time the abscess on the left side steadily healed. The active suppurating condition on the right side demanded further attention. Under ether,

March 4, 1897, I made an incision above the opening of the sinus, carrying it down to and encircling its mouth and extending it well toward the groin. Entering the abdominal cavity above the sinus, guided by my index finger, I freed the adhesions, separated the skin, protected the abdominal cavity by pads and ultimately reached the suppurating area below. The mouth of the sinus was, of course, packed temporarily with a strip of iodoform gauze. A small stream of pus was seen to find its way into the abscess cavity from above. Following this up I entered a large post-cecal abscess filled with thick yellow pus. This was likewise freely opened and cleansed. Freeing the cecum I was soon able to reach the appendix. This we removed, closing over the stump with a cuff of serous membrane. Gauze drainage was generously used. Some was carried quite deeply. Having curetted away all granulation tissue the wound was closed in part.

Everything progressed favorably with him until the third day after the operation, when he presented marked distension, great abdominal distress, dry tongue and bad facial expression; pulse ranged between 110 and 120; the cecum was being forced out of the wound. He continued very sick throughout the night. The next morning he was given epsom salts, which only precipitated the vomiting of a great deal of bilious matter. There was no fecal discharge from the bowels, but a great deal of gas and mucus passed from him. The greatest distension was in the hypogastric, umbilical and right iliac regions. About this time a small quantity of pus was noticed forcing its way up out of the granulations from the wound on the left side. This wound had been as stated, all but healed. With the aid of a narrow bladed curved forceps I succeeded in carrying a drainage-tube into the sinus on the left side for a distance of about nine inches and immediately there was a free discharge of pus.

The patient soon improved. The symptoms of intestinal obstruction disappeared. His pulse became firm, reduced to 100; temperature to normal; bowels moved and general improvement was apparent. The abdomen became flat; distension not so marked; tongue was moist; and the stomach no longer made itself unpleasantly manifest. This improvement continued for about two weeks. The cecum, which from the very beginning of his sickness was extensively involved, occasioned each day more difficulty. The hospital interne who made the dressings complained to me of his fear that the gut would ultimately become perforated at this point.

On the 28th day of March I was informed by the interne that a perforation had occurred. The opening was very small and I hoped that we might be able to close it with Lembert suture, under cocaine. This was attempted, but on April 2d it again gave way and a few days later there was quite a free fecal discharge from the wound. Throughout the month of April, however, while some fecal masses were daily discharged through the fecal fistula, most of his feces were expelled per rectum.

The sinus on the left side steadily discharged less, and by May 1st was entirely healed. During the month of May the fecal fistula became gradually converted into an artificial anus and from this time forward there was very little discharge by the natural channel. The post-cecal abscess cavity filled in very slowly and only partially. During the summer months, being out of doors and possessing a good appetite, he gained in strength and health. The artificial anus was guarded by a receptacle. Once during the month of June there appeared some fever, with its ordinary accompanying manifestations, together with a large firm area in the center of the abdomen just below the umbilicus. It was quite immovable and very tender. With the aid of high enemata, cathartics and free irrigation of the wound cavity, this cleared up after a few weeks.

Returning from my vacation early in September, I found him in good physical condition, but with his artificial anus still the point of exit of his entire fecal discharge. The mucous membrane was strongly everted and the opening into the cecum was large enough to receive two fingers.

On the 19th of September, a little more than a year after his primary operation, I undertook what I believe will be the final operation for the relief of his condition. The necessary preparations, including thorough emptying of the intestinal tract completed, I made an incision, beginning about three inches above the artificial anus, entering the abdomen at this point, cutting down to and around the artificial anus, and downward toward the groin. Protecting the peritoneal cavity as I went, by gauze pads, I gradually severed the adherent intestine along its inner line of attachment. The difficulty came in separating the adhesions to its outer surface. These were so firm and the cecal wall so degenerated that at one point the bowel was badly torn open, so that we had not only the preëxisting opening, but another fully two inches in length. All of the ragged, thickened, friable portion of the cecum was cut away and the two wounds closed by double Lembert sutures. The size of the gut was greatly reduced. The ileo-cecal opening seemed quite small and the structures in its neighborhood were deeply infiltrated. The gut was securely closed, freed from all of its adhesions, and the post-cecal abscess cavity packed with gauze and the wound closed in part. At this as well as at all of his previous operations the anesthetic gave us a great deal of trouble. At each the patient stopped breathing and required artificial respiration to restore him. The reactionary temperature following this operation was 101°. On the second day it was normal, his pulse fell below 90, and both have remained so since. Some of the discharge during the first week had a very suspicious odor, probably from the osmosis of gases, but at no time was there any fecal leakage.

As is my practice, the wound has been closed little by little by the introduction of secondary sutures, until we have now a wound of about two-thirds of an inch in length, while in depth the post-cecal space is all but filled, and there is not the slightest suggestion of the projection of the intestines into sight. The abdomen has remained flat throughout—there has been scarcely any bloating. Bowels move freely and sufficiently. He sits up; eats what he pleases. There is no further discharge from the wound, and the period of his ultimate recovery can now be predicted to be close at hand.

The case presents several very interesting points: (1) The extensive general peritonitis, with the accompanying profound sepsis, would hardly have warranted a prediction of even possible recovery after the primary operation; (2) When four months after the occurrence of the appendicitis he became my patient, I hoped that the large fecal concretion in the sinus was alone responsible for its unhealed condition. The accompanying left-sided abscess was first believed to be due to a localised infection growing out of the widespread general peritonitis. Not until the operation done for the removal of the appendix, and the finding at that time of a large post-cecal abscess, did connection between the suppurating conditions of the two sides suggest itself to me. But, when I cut off the exit of pus on the right side by my gauze packing, and thereby forced any purulent collection in its depth to find another outlet, I unwittingly reopened the communication with the abscess on the left and proved beyond question the existence of a communicating sinus, extending across the whole length of the retroperitoneal space.

I have gone over the surgical literature of recent years quite carefully and must confess that I have not found another case presenting an exactly similar condition. I have seen appendicular abscesses allowed to open spontaneously, in which the pus has found an exit through the umbilicus, the scrotum, and several in the lumbar region, strikingly simulating perinephritic abscess. I know there are cases on record where the pus, of appendicular origin, has found a way through the sub-diaphragmatic space, broken into the lung and been expectorated. I have found another case recorded where the transit of pus was through the abdominal wall and thus out on the left side. But none quite like my case. (3) While it is no unusual thing to meet with a fecal discharge, because of perforation after operation for appendicular affections, the formation of an artificial anus is unusual. As we are told by Dr. Elsner that at the primary operation the cecum was all but gangrenous, we can readily appreciate its very friable condition and the little vitality it manifested later on. Fortunately there is plenty of tissue to spare in the cecum, and one can cut away generous strips from it without fear. The attempt to close the fecal fistula without separating the gut freely was, as might have been expected, futile. But now, after more than a year's sickness and a number of operative procedures, we have finally reached a happy termination in what has been to me a case of appendicitis with most unusual sequelæ.

430 South Salina Street.

